# Targeted Proteomics Reveals Inflammatory Pathways that Classify Immune Dysregulation in Common Variable Immunodeficiency

**DOI:** 10.1007/s10875-020-00908-1

**Published:** 2020-11-15

**Authors:** Roos-Marijn Berbers, Julia Drylewicz, Pauline M. Ellerbroek, Joris M. van Montfrans, Virgil A. S. H. Dalm, P. Martin van Hagen, Baerbel Keller, Klaus Warnatz, Annick van de Ven, Jaap M. van Laar, Stefan Nierkens, Helen L. Leavis

**Affiliations:** 1 Department of Rheumatology & Clinical Immunology, University Medical Center Utrecht and Utrecht University, Heidelberglaan 100, 3584 CX Utrecht, The Netherlands; 2 Center for Translational Immunology, University Medical Center Utrecht and Utrecht University, Utrecht, The Netherlands; 3 Department of Internal Medicine and Infectious Diseases, University Medical Center Utrecht and Utrecht University, Utrecht, The Netherlands; 4 Department of Pediatric Immunology and Infectious Diseases, University Medical Center Utrecht and Utrecht University, Utrecht, The Netherlands; 5 Department of Internal Medicine, Division of Clinical Immunology; Department of Immunology; Academic Center for Rare Immunological Diseases (RIDC), Erasmus University Medical Center Rotterdam, Rotterdam, The Netherlands; 6 Department of Rheumatology and Clinical Immunology, Medical Center - University of Freiburg, Faculty of Medicine, University of Freiburg, Freiburg, Germany; 7 Center for Chronic Immunodeficiency (CCI), Medical Center - University of Freiburg, Faculty of Medicine, University of Freiburg, Freiburg, Germany; 8 Departments of Internal Medicine and Allergology, Rheumatology and Clinical Immunology, University Medical Center Groningen, Groningen, The Netherlands

**Keywords:** Immune dysregulation, Common variable immunodeficiency (CVID), Cytokines, Biomarkers, Prediction, Primary immunodeficiency

## Abstract

Patients with common variable immunodeficiency (CVID) can develop immune dysregulation complications such as autoimmunity, lymphoproliferation, enteritis, and malignancy, which cause significant morbidity and mortality. We aimed to (i) assess the potential of serum proteomics in stratifying patients with immune dysregulation using two independent cohorts and (ii) identify cytokine and chemokine signaling pathways that underlie immune dysregulation in CVID. A panel of 180 markers was measured in two multicenter CVID cohorts using Olink Protein Extension Assay technology. A classification algorithm was trained to distinguish CVID with immune dysregulation (CVIDid, *n* = 14) from CVID with infections only (CVIDio, *n* = 16) in the training cohort, and validated on a second testing cohort (CVIDid *n* = 23, CVIDio *n* = 24). Differential expression in both cohorts was used to determine relevant signaling pathways. An elastic net classifier using MILR1, LILRB4, IL10, IL12RB1, and CD83 could discriminate between CVIDid and CVIDio patients with a sensitivity of 0.83, specificity of 0.75, and area under the curve of 0.73 in an independent testing cohort. Activated pathways (fold change > 1.5, FDR-adjusted *p* < 0.05) in CVIDid included Th1 and Th17-associated signaling, as well as IL10 and other immune regulatory markers (LAG3, TNFRSF9, CD83). Targeted serum proteomics provided an accurate and reproducible tool to discriminate between patients with CVIDid and CVIDio. Cytokine profiles provided insight into activation of Th1 and Th17 pathways and indicate a possible role for chronic inflammation and exhaustion in immune dysregulation. These findings serve as a first step towards the development of biomarkers for immune dysregulation in CVID.

**Supplementary Information:**

The online version contains supplementary material available at 10.1007/s10875-020-00908-1.

## Introduction

Common variable immunodeficiency disorder is a primary immunodeficiency hallmarked by low serum immunoglobulins and impaired production of specific antibodies in response to vaccinations, resulting in increased risk for recurrent bacterial infections [1, 2]. The cause of CVID is assumed to be multifactorial with estimated heritability around 20% [3], and while new monogenetic causes are discovered each year, in the majority of cases a genetic cause remains undefined [4]. Under adequate immunoglobulin replacement therapy (IgRT) severe infections can usually be prevented, but over a third of patients develop additional complications related to immune dysregulation [4–6]. These complications include autoimmune disease, granuloma formation, lymphoproliferative disease including increased risk of lymphoma, and enteropathy [7], and together they cause most morbidity and mortality in CVID [4, 5]. There is no consensus about how to treat inflammatory complications in CVID, with expert opinion-based guidelines varying per country [8, 9]. The identification of predictive biomarkers for immune dysregulation, and biomarkers that can be used to monitor therapeutic response are needed to improve clinical care for these patients. In addition, better understanding of the underlying immune mechanisms that cause immune dysregulation can provide new therapeutic targets and aid in better selection of novel targeted immunotherapies such as cytokine blockade or JAK/STAT inhibitors in the clinic.

Known risk factors for the development of immune dysregulation in CVID include low naïve CD4 T cells [10], increased peripheral CD21^low^ B cells [11, 12], and IgA deficiency [10, 12]. These factors predict long-term risk to develop clinical complications, but do not inform about a current inflammatory state, and are therefore less suitable to monitor short-term disease progression or therapeutic response. For those purposes, the use of serum cytokine and chemokine biomarkers is entering clinical practice in other inflammatory diseases, for example soluble IL2 receptor in hemophagocytic lymphohistiocytosis [13], CXCL10 in juvenile dermatomyositis [14], and IL18 in adult-onset Still’s disease and systemic juvenile idiopathic arthritis [15].

Previous studies in CVID have frequently reported conflicting results about serum cytokines (reviewed by Varzaneh et al. [16]). For example, IL10 is often found to be upregulated in CVID as compared to healthy controls [17, 18], but in a different cohort, a decrease of serum IL10 was described [19]. Overall, markers associated with an activated myeloid compartment are consistently upregulated in CVID [18–20], and the T-helper (Th) serum cytokine profile observed in immune dysregulation in CVID is mostly found to be Th-1 driven [17, 18, 21]. Varying findings in these studies highlight the need to consider CVID patients with immune dysregulation (CVIDid) separately from patients with an “infection only” (CVIDio) -phenotype.

As a first step to the identification of biomarkers for immune dysregulation in CVID, we used a targeted proteomic approach in two clinically diverse multicenter CVID cohorts in order to (i) assess the potential of serum proteomics in stratifying patients with immune dysregulation from patients with infections only using two independent cohorts and (ii) identify cytokine and chemokine signaling pathways that underlie immune dysregulation in CVID.

## Materials and Methods

### Ethics Statement

Ethical approval for this study for all Dutch participants was received from the Medical Ethical Committee of the Erasmus Medical Centre in Rotterdam, the Netherlands (METC: NL40331.078). Ethical approval for sampling of patients included from Freiburg, Germany was received from the University of Freiburg Ethics Committee 282/11. Written informed consent was obtained from all patients and controls according to the Declaration of Helsinki.

### Study Population and Sample Collection

Patients diagnosed with common variable immunodeficiency disease according to the European Society for Immunodeficiencies criteria [1] aged 7 or older were included during outpatient clinic visits of the University Medical Center Utrecht, the Erasmus Medical Center in Rotterdam and the University Medical Center Groningen, in the Netherlands, and the University Medical Center Freiburg, Germany. Healthy controls (HC) were recruited from household members of patients. Medication use up to 3 months prior to sampling was recorded. Clinical characteristics were collected from electronic patient files.

### Targeted Proteomics

Serum samples were stored at – 80 °C within 4 h of sampling until use. Serum levels of 180 unique inflammation and immune response–related proteins (supplementary Table S1) were measured using proximity extension immunoassay (PEA; Olink Proteomics, Uppsala, Sweden) [22], using the ProSeek Multiplex Inflammation and Immune Response kits.

Briefly, the proximity extension immunoassay technology is based on dual recognition of serum proteins by pairs of antibodies coupled to a cDNA-strand that ligates when brought into proximity by its target. This DNA tag is PCR amplified and detected using a Biomark HD 96 × 96 dynamic PCR array (Fluidigm, San Francisco, USA). After corrections from DNA extension- and interpolation controls, a normalized protein expression value (NPX) is generated on a log-2 scale.

### Data Analysis and Statistics

All data analysis was performed in R (version 3.5.1) [23], and all scripts used have been made publicly available on https://gitlab.com/rberbers/cvid_cytokines_olink.

PEA data was analyzed using NPX values (on a log2 scale) unless stated otherwise. For proteins that were detected below the lower limit of detection (LLOD), the measured value was replaced by the LLOD value/2. The healthy control samples (training cohort *n* = 15, testing cohort *n* = 27) were used to correct for batch effects between the first and the second cohort.

PCA was performed using the package *prcomp*, and 3D plots displaying the first three principal components were generated using the packages *rgl* and *car*. Differences between the clinical groups were assessed using pairwise PERMANOVA with correction for false discovery rate (FDR) using the packages *vegan* and *pairwiseAdonis*. Volcano plots were generated using Mann–Whitney *U* tests with Benjamini-Hochman correction for FDR and log2 fold change calculated on the linear scale (2^^NPX^). Differential expression was defined as FDR-adjusted (adj.) *p* < 0.05 and log2 fold change > 0.58 (equal to linear fold change of > 1.5).

For the classification model, the following machine learning algorithms were tuned on the training cohort using the package *caret*: random forest (package *randomForest*), elastic net regression (package *glmnet*) and extreme gradient boosting (package *xgboost*), with fivefold repeated crossvalidation. All measured biomarkers with the addition of age and sex were included in the training of the algorithms. Prediction performance of the final model on the testing cohort was assessed with area under the curve (AUC) of receiver-operator curves (ROC) using the R-package *pROC*. The sensitivity and specificity for each model was calculated for the threshold with maximum Youden’s Index (sensitivity+specificity − 1). Regression coefficients of selected variables were standardized by their standard deviation.

Pathway analysis was performed using Ingenuity Pathway Analysis (IPA) software (QIAGEN Inc., https://www.qiagenbioinformatics.com/products/ingenuity-pathway-analysis) [24], using differentially expressed proteins detected in the volcano plots and the 180 measured proteins as the reference dataset.

## Results

### Exploratory Analysis of the Serum Protein Profile of CVID with Immune Dysregulation, CVID with Infection Only, and Healthy Controls

In order to evaluate whether serum cytokine profiles could be used to distinguish CVIDio from CVIDid, 180 serum markers (supplementary Table S1) were measured in two independent cohorts using a proximity extension assay. Patients were categorized as CVIDid when they had clinical (history of) autoimmune disease, GLILD, granulomatous disease other (nonGLILD), lymphoproliferation, enteritis, and/or malignancy. Splenomegaly was also recorded but splenomegaly alone was not sufficient to be categorized as CVIDid. Patients without any of these complications were classified as CVIDio. All CVID patients were on IgRT at the time of sampling.

First, an age- and gender-balanced training cohort (Table 1) was selected using 45 participants (15 healthy controls (HC), 16 CVIDio, and 14 CVIDid) from two academic hospitals in the Netherlands. The most common clinical complication in the CVIDid group was autoimmune disease, (64%), followed by and enteritis (35%). Splenomegaly was also highly prevalent at 50% of CVIDid patients in this cohort. Genetic screening had been performed for clinical care in a minority of patients (6% of CVIDio and 35% CVIDid), yielding one patient with CTLA4 haploinsufficiency, one patient with STAT1 gain-of-function, and two patients with variants of unknown significance (VUS) in the CVIDid group (supplementary Table S2); one patient with a VUS in UNC13D and one patient with VUS in PLCG2 and heterozygosity for JAK3. In the CVIDio group, one TNFRSF13B (TACI) mutation was found.

**Table 1:** Characteristics training cohort. *types of autoimmune disease: monoarthritis, rheumatoid arthritis, coeliac disease, Sjögren’s disease, autoimmune (thrombo-)cytopenias, alopecia, vitiligo, myositis

	HC	CVIDio	CVIDid
Total *N*	15	16	14
Age (years), median (IQR)	38 (35–57)	38.5 (28.25–50.5)	41.5 (34–51.75)
Male *N* (%)	7 (47%)	8 (50%)	7 (50%)
Inclusion site *N* (%)			
Utrecht, the Netherlands	9 (60%)	11 (69%)	10 (71%)
Rotterdam, the Netherlands	6 (40%)	5 (31%)	4 (29%)
Clinical phenotype *N* (%)			
AI disease	0	0	9 (64%) *
GLILD	0	0	2 (14%)
Granulomatous disease other	0	0	2 (14%)
Enteritis	0	0	5 (35%)
Lymphoproliferation	0	0	1 (7%)
Malignancy	0	0	2 (14%)
Splenomegaly	0	0	7 (50%)
Medication use during 3 months prior to sampling *N* (%)			
Antibiotics	0	0	4 (29%)
Immune suppressive therapy	0	0	0
Genetics N (%)			
Genetics done	0	1 (6%)	5 (35%)
Nothing found	0	0	1 (7%)
VUS found	0	0	2 (14%)
Relevant pathogenic mutation found	0	1 (6%)	2 (14%)

Next, 74 participants (27 HC, 24 CVIDio and 23 CVIDid) from four academic hospitals in the Netherlands and Germany were included in a second independent testing cohort (Table 2). In this cohort, there were more males in the CVIDid group (70% in CVIDid vs 33% in HC and 46% in CVIDio), and the CVIDid group was younger (mean age 36.7 in CVIDid, 40.1 in CVIDio, 44.3 in HC). The most common clinical complications in the CVIDid group were autoimmune disease (57%) and enteritis (43%), similar to prevalence in the training cohort. Four patients received immunosuppressive therapy around time of sampling; two patients used TNF-α blockade, and two prednisone (5 mg and 40 mg/day, respectively). In this cohort, 30% of CVIDid and 29% of CVIDio patients had been genetically screened (supplementary Table 2), resulting in three TNFRSF13B mutations and one PIK3R1 mutation found in the CVIDid group. No relevant mutations were found in CVIDio.

**Table 2:** Characteristics testing cohort. *types of autoimmune disease: systemic lupus erythematosus-like disease, Sjögren’s disease, autoimmune (thrombo-)cytopenias, type 1 diabetes, membranous glomerulonephritis, alopecia, hepatitis, vitiligo

	HC	CVIDio	CVIDid
Total *N*	27	24	23
Age (years), median (IQR)	43 (37–49)	37 (25–57.25)	37 (23–49)
Male *N* (%)	9 (33%)	11 (46%)	16 (70%)
Inclusion site *N* (%)			
UMCU	19 (70%)	11 (46%)	13 (57%)
EMC	4 (15%)	7 (29%)	3 (13%)
UMCG	4 (15%)	0	0
Freiburg	0	6 (24%)	7 (30%)
Clinical phenotype *N* (%)			
AI disease	0	0	13 (57%)
GLILD	0	0	8 (35%)
Granulomatous disease other	0	0	1 (4%)
Enteritis	0	0	10 (43%)
Lymphoproliferation	0	0	6 (26%)
Malignancy	0	0	1 (4%)
Splenomegaly	0	0	10 (43%)
Medication use during 3 months prior to sampling *N* (%)			
Antibiotics	0	8 (33%)	5 (22%)
Immune suppressive therapy	0	0	4 (17%)
**Genetics** ***N*** **(%)**			
Genetics done	0	7 (29%)	7 (30%)
Nothing found	0	7 (29%)	2 (9%)
VUS found	0	0	1 (4%)
Relevant pathogenic mutation found	0	0	4 (17%)

**Fig. 1:**
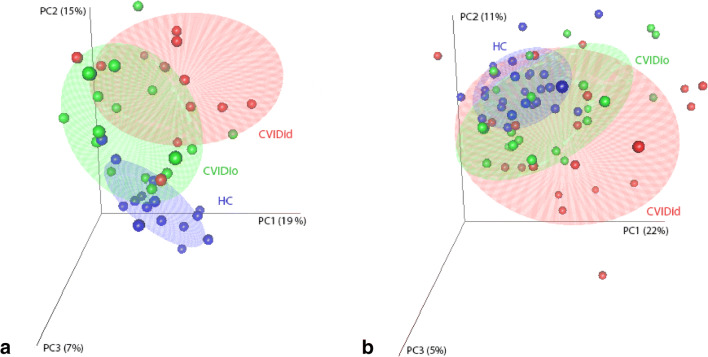
Principal component analysis of first 3 principal components (PC). Ellipses indicate 95% confidence intervals. **a** Training cohort. CVIDid: CVID with immune dysregulation (*n* = 14), CVIDio: CVID with infection only (*n* = 16), HC: healthy controls (*n* = 15). FDR-corrected pairwise PERMANOVA using Euclidean distance: CVIDid vs CVIDio adj. *p* = 0.003; CVIDid vs HC adj. *p* = 0.002; CVIDio vs HC adj. *p* = 0.002. **b** Testing cohort: CVIDid (*n* = 23), CVIDio (*n* = 24), HC (*n* = 27). FDR-corrected pairwise PERMANOVA using Euclidean distance: CVIDid vs CVIDio adj. *p* = 0.144; CVIDid vs HC adj. *p* = 0.003; CVIDio vs HC adj. *p* = 0.005

A total of 180 unique inflammation- and immune response–related proteins were quantified in serum of the training and testing cohort. Principal component analysis (PCA) shows distinct clustering of HC, CVIDio and CVIDid patients in the training cohort (Fig. 1a, CVIDid vs CVIDio adj. *p* = 0.003, CVIDid vs HC adj. *p* = 0.002, CVIDio vs HC adj. *p* = 0.002). One outlier in the CVIDid group was the patient with a heterozygote mutation in JAK3 and a VUS in PLCG2. In the testing cohort (Fig. 1b), PCA showed significant clustering of CVIDid and CVIDio from HC (CVIDid vs HC adj. *p* = 0.003, CVIDio vs HC adj. *p* = 0.005), but there was more overlap between CVIDid and CVIDio in this cohort (CVIDid vs CVIDio adj. *p* = 0.144), with larger spread of the CVIDid group.

### Machine Learning Approaches Reveal a Serum Protein Signature Consisting of MILR1, LILRB4, IL10, IL12RB1, and CD83 to Classify Immune Dysregulation in CVID

In order to assess whether the serum protein profiles could be used to classify immune dysregulation in CVID, three machine learning algorithms (random forest, elastic net, and extreme gradient boosting) were trained on the training cohort, and their performance was assessed by using the resulting models to predict which patients had immune dysregulation in the second independent testing cohort. Given the clinical context in which detection of patients at risk for immune dysregulation is desirable, a high sensitivity was deemed more important than a high specificity.

**Fig. 2:**
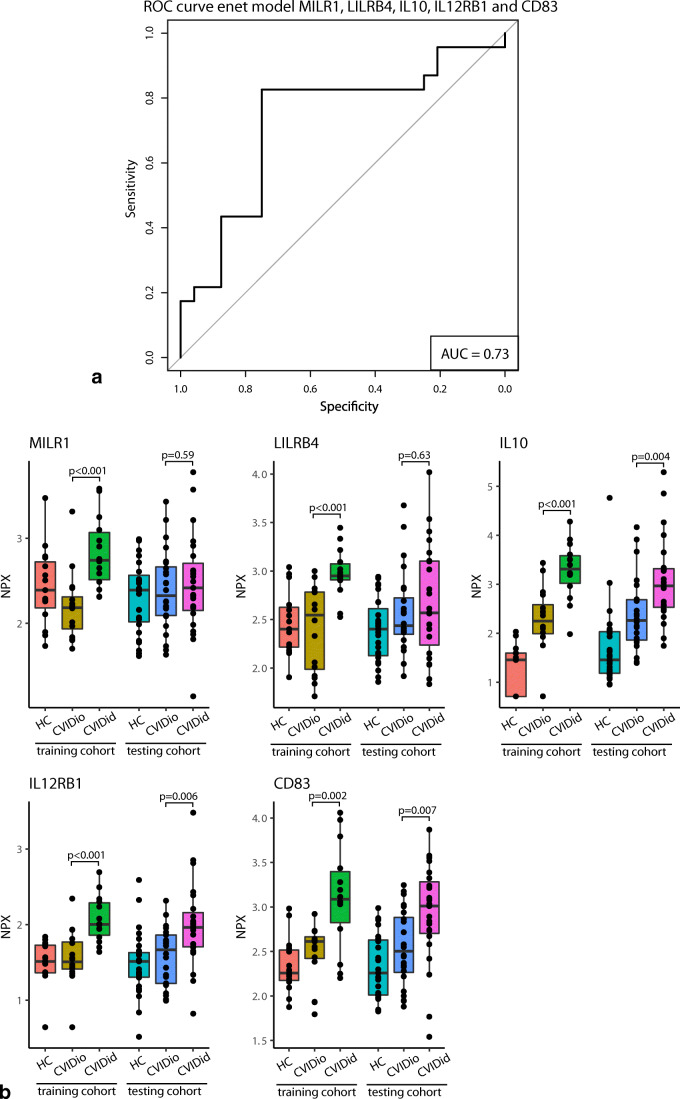
**a** Receiver-operator curve (ROC) for the classification of CVIDid vs CVIDio on the testing cohort (CVIDid *n* = 23, CVIDio *n* = 24) using the elastic net model using MILR1, LILRB4, IL10, IL12RB1 and CD83 as variables. **b** Distribution of classifying variables selected in the elastic net model. Training cohort healthy controls (HC, *n* = 15), CVIDio (*n* = 16), CVIDid (*n* = 14), testing cohort HC (*n* = 27), CVIDio (*n* = 24), CVIDid (*n* = 23). The horizontal line inside the box represents the median. The whiskers represent the lowest and highest values within 1.5 × interquartile range. *P* values: Mann–Whitney *U* test after FDR correction

Elastic net (enet) and extreme gradient boosting (xgb) were the best performing algorithms (supplementary Table S3). In order to reduce overfitting on the training cohort, only the markers that were selected as the most important variables by both models were selected for the final algorithm: mast cell immunoglobulin-like receptor 1 (MILR1), leukocyte immunoglobulin-like receptor subfamily B member 4 (LILRB4), IL10, IL12 receptor subunit beta 1 (IL12RB1), and CD83 (an immunoglobulin superfamily receptor expressed by mature antigen presenting cells [25]). The elastic net model using these five proteins yielded the best combination of high AUC and sensitivity: AUC of 0.73, sensitivity of 0.83 and specificity of 0.75 with threshold selected for maximum Youden’s Index (Fig. 2a and Table 3). Despite being trained only on samples collected in the Netherlands, this model performed equally well on the samples from the testing cohort collected in Freiburg (Germany), correctly identifying 12/13 samples. The two patients receiving TNF-α blockade were grouped correctly as CVIDid, but the patient who was sampled under 40-mg prednisone was misclassified as CVIDio.

**Table 3:** performance of the enet model using MILR1, LILRB4, IL10, IL12RB1 and CD83 as classifiers. Classification predicted at threshold with maximum Youden’s Index

		True		
		CVIDid	CVIDio	Total predicted
Predicted	CVIDid	19	6	25
	CVIDio	4	18	22
	Total true	23	24	

While IL10 (training set *p* < 0.001, testing set *p* = 0.004), IL12RB1 (training set *p* < 0.001, testing set *p* = 0.006) and CD83 (training set *p* = 0.002, testing set *p* = 0.007), were consistently significantly upregulated in CVIDid compared to CVIDio in both the training and the testing cohort (Fig. 2b), this was not the case for MILR1 and LILRB4. These two were significantly upregulated in the training cohort (*p* < 0.001 for both) but not in the testing cohort (*p* = 0.59 and *p* = 0.63, respectively), so the performance of the model without these markers was assessed in a post hoc analysis (supplementary Table S4). A logistic regression model using only IL10, IL12RB1 and CD83 yielded a higher AUC (0.76) than the original model, but with lower sensitivity (0.79) specificity (0.71) at maximum Youden’s Index on the testing cohort.

### Immune Dysregulation in CVID Is Characterized by Upregulation of T Helper 1, T Helper 17, and Immune Regulatory Proteins

**Fig. 3:**
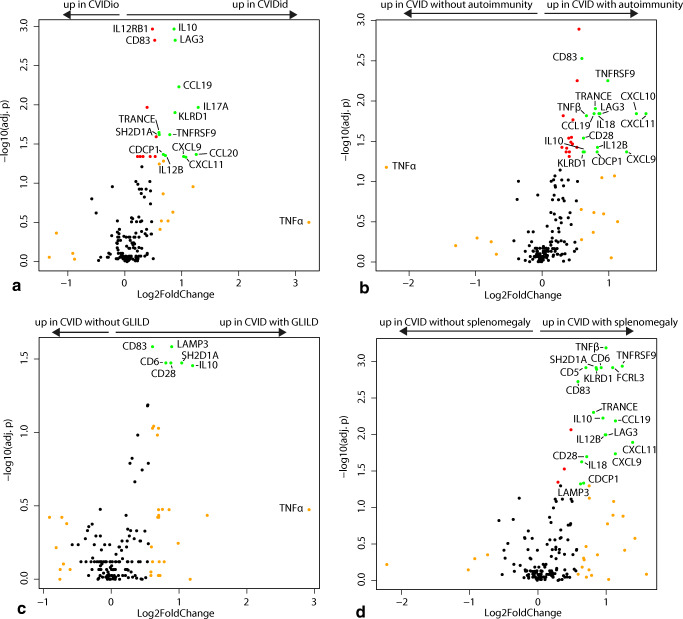
Volcano plots: Green dots indicate markers with log2 fold change > 0.58 (= fold change 1.5) and FDR-adjusted *p* value < 0.05. Red dots indicate markers with log2 fold change < 0.58 and FDR-adjusted *p* value < 0.05. Orange dots indicate markers with log2 fold change > 0.58 and FDR-adjusted *p* value > 0.05. **a** Differential expression analysis of proteins upregulated in CVID with immune dysregulation (CVIDid, *n* = 37) as compared to CVID with infection only CVIDio, *n* = 40). **b** Differential expression analysis of proteins upregulated in CVID with autoimmunity (*n* = 22) as compared to CVID without autoimmunity (*n* = 55). **c** Differential expression analysis of proteins upregulated in CVID with GLILD (*n* = 10) as compared to CVID without GLILD (*n* = 67). **d** Differential expression analysis of proteins upregulated in CVID with splenomegaly (*n* = 17) as compared to CVID without splenomegaly (*n* = 60)

In order to infer which inflammatory pathways were differentially expressed in immune dysregulation in CVID, the training and testing cohorts were merged. In CVIDid (*n* = 37), thirteen proteins were differentially upregulated compared to CVIDio (*n* = 40) with FDR-corrected *p* value < 0.05 and fold change (FC) > 1.5 (Fig. 3a, supplementary fig. S1A). These markers included cytokine IL10 (adj. *p* = 0.001, FC = 1.82) and receptors LAG3 (adj. *p* = 0.001, FC = 1.85) and TNFRSF9 (also known as 4-1BB, adj. *p* = 0.024, FC = 1.73), all associated with negative regulation of the immune response. Th1 activation was observed in the upregulation of CXCL9 (adj. *p* = 0.046, FC = 2.05) and CXCL11 (adj. *p* = 0.047, FC = 2.10). In addition, cytokines and chemokines associated with Th17 activation were upregulated, including IL17A (adj. *p* = 0.011, FC = 2.44), IL12B (also known as IL12p40, a subunit of IL12 and IL23; adj. *p* = 0.044, FC = 1.64) and mucosal tissue homing chemokine CCL20 (adj. *p* = 0.043, FC = 2.39). IL-6 production, which would be consistent with activated Th17 cells, was upregulated in both CVIDid (adj. *p* < 0.001, FC = 2.34) and CVIDio (adj. *p* = 0.001, FC = 2.14) compared to HC, but not between CVIDid and CVIDio (adj.*p* = 0.58, FC = 1.09) (data not shown).

Natural killer (NK) cell activation marker KLRD1 (adj. *p* = 0.013, FC = 1.84) was upregulated in CVIDid and CVIDio, as well as SH2D1A (adj. *p* = 0.024, FC = 1.52), which is involved in NK- T- and B cell stimulation. Also upregulated in CVIDid were TRANCE (also known as RANK-L, adj. *p* = 0.023, FC = 1.52) which induces monocyte chemotaxis, and CCL19 (adj. *p* = 0.006, FC 1.93) which induces lymphocyte homing to secondary lymphoid organs.

One outlier in this analysis was TNF-α, which was not significantly upregulated (adj. *p* = 0.316) but had a high fold change (FC = 9.41) (supplementary fig. S2). This was driven by the two patients who used TNF-α blockade therapy and had high serum levels of TNF-α, an effect that has previously been observed [26]. These two patients did not have aberrant expression of other proteins and therefore were not excluded from the study (data not shown). TNF-α levels excluding these two patients were not different between CVIDid and CVIDio (adj. *p* = 0.46, FC = 1.18), but were upregulated in CVIDid (adj. *p* = 0.0005, FC = 1.45) and CVIDio (adj.*p* = 0.003, FC = 1.23) compared to HC.

### Autoimmune Disease, GLILD, and Splenomegaly in CVID Are Associated with a Distinct Serum Protein Profile

CVID with autoimmune disease (*n* = 22) was characterized by upregulation of fifteen proteins as compared to CVID without autoimmune disease (*n* = 55) (Fig. 3b, supplementary fig. S1B), with much overlap with the upregulated proteins observed in CVIDid. In this subgroup analysis, cytokines and chemokines associated with Th1 signaling were upregulated: CXCL9 (adj. *p* = 0.043, FC = 2.41), CXCL10 (adj. *p* = 0.014, FC = 2.95), CXCL11 (adj. *p* = 0.014, FC = 2.67), IL18 (adj. *p* = 0.014, FC = 1.81), and CD28 (adj. *p* = 0.029, FC = 1.54). Markers of negative immune regulation were also increased in autoimmunity: LAG3 (adj. *p* = 0.014, FC = 1.82) and TNFRSF9 (adj. *p* = 0.006, FC = 1.98), and CD83 (adj. *p* = 0.003, FC = 1.51) which in soluble form is thought to be immunosuppressive [27].

In CVID with GLILD (*n* = 10) (Fig. 3c, supplementary fig. S1C), negative regulators CD83 (adj. *p* = 0.026, FC = 1.53) and IL10 (adj. *p* = 0.035, FC = 2.30) were again upregulated as compared to CVID without GLILD (*n* = 67). In addition, LAMP3 (adj. *p* = 0.026, FC = 1.86) was upregulated, which is associated with DC maturation and is especially highly expressed in type-2 pneumocytes in the lung [28]. T cell receptor coreceptors CD6 (adj. *p* = 0.034, FC = 1.84) and CD28 (adj. *p* = 0.034, FC = 1.75) were also upregulated in GLILD.

In CVID patients with splenomegaly (*n* = 17) (Fig. 3d, supplementary fig. S1D), nineteen markers were upregulated compared to CVID without splenomegaly (*n* = 60). These included markers of immune suppression, such as TNFRSF9 (adj. *p* = 0.001, FC = 2.36), CD83 (adj. *p* = 0.002, FC = 1.50), LAG3 (adj. *p* = 0.010, FC = 1.99), IL10 (adj. *p* = 0.006, FC = 1.94), and FC receptor-like protein 3 (FCRL3) (adj. *p* = 0.001, FC = 2.14) [29]. Increased Th1-associated proteins were CXCL9 (adj. *p* = 0.018, FC = 2.20), CXCL11 (adj. *p* = 0.013, FC = 2.62), IL18 (adj. *p* = 0.024, FC = 1.56), CD28 (adj. *p* = 0.020, FC = 1.64) and IL12B (adj. *p* = 0.010, FC = 1.98). Inflammatory markers KLRD1 (adj. *p* = 0.001, FC = 1.80), TNF-β (adj. *p* < 0.001, FC = 2.00), SH2D1A (adj. *p* = 0.001, FC = 1.82), CD5 (adj. *p* = 0.001, FC = 1.63), CD6 (adj. *p* = 0.001, FC = 1.90) and one of its ligands CDCP1 (adj. *p* = 0.048, FC = 1.54), and TRANCE (adj. *p* = 0.005, FC = 1.76) were also upregulated.

Subgroup analyses of granulomatous disease, lymphoproliferation, enteritis, and malignancy did not yield any differentially expressed proteins, possibly due to smaller sample size**.**

**Fig. 4:**
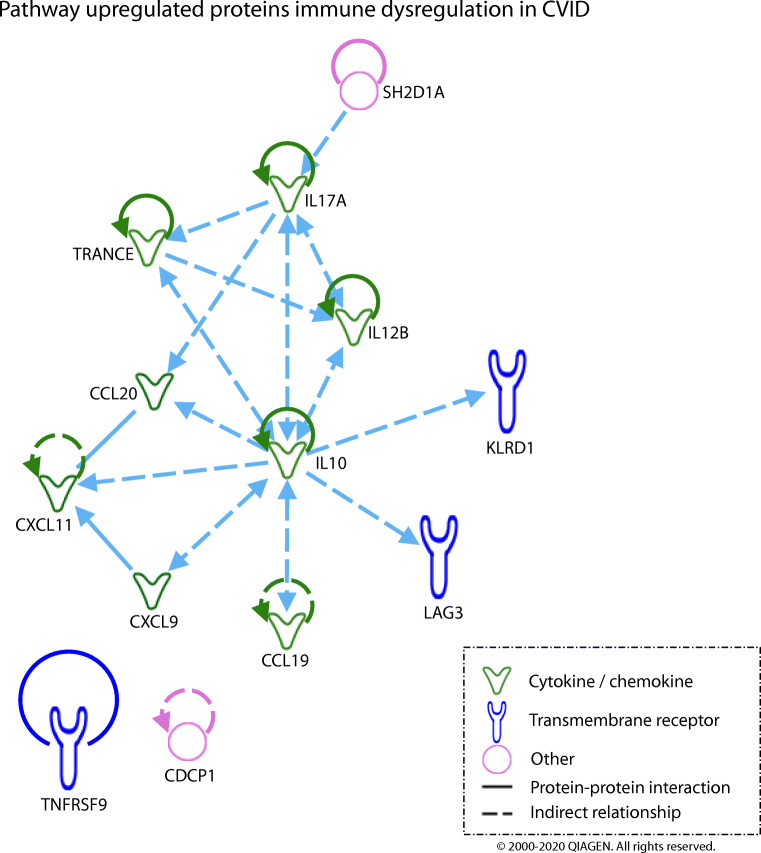
Literature-based pathway analysis of differentially expressed proteins upregulated in CVID with immune dysregulation (CVIDid) as compared to CVID with infections only (CVIDio), using ingenuity pathway analysis software

In order to integrate these findings, a literature-based pathway analysis was performed using ingenuity pathway analysis. Of the upregulated proteins in CVIDid, IL10 had the most connections to the other differentially expressed markers (Fig. 4a), indicating that IL10 may be a keystone regulator in the inflammatory profile of CVIDid. The same was observed for pathway analysis of proteins upregulated in autoimmunity, GLILD, and splenomegaly (data not shown).

## Discussion

We demonstrated that an algorithm using serum biomarkers MILR1, LILRB4, IL10, IL12RB1, and CD83 identified by targeted serum proteomics could classify immune dysregulation in CVID in a discovery cohort and in our independent testing cohort, providing a first step towards the development of a screening tool for immune dysregulation in CVID. Of the selected biomarkers, IL10, IL12RB1, and CD83 were consistently upregulated in the testing and the training cohorts, in contrast to MILR1 and LILRB4 which were not reproducible in the testing cohort. This may be due to sensitivity of MILR1 and LILRB4 to batch effects or minute differences in sample handling. However, an algorithm using only IL10, IL12RB1, and CD83 performed almost as well as when MILR1 and LILRB4 were included. This is in accordance with previous findings of upregulated IL10 [17, 18, 21], IL12 [18, 21], and CD83 [21] in CVID compared to healthy controls.

As a next step towards the application of this screening tool, the dynamics of these serum markers in early disease need to be monitored, as this cohort included only patients with current immune dysregulation or immune dysregulation in remission. If these markers are upregulated before the full clinical phenotype has developed, the algorithm may be used for early detection of disease, and allow for earlier intervention with immunosuppressive therapy. Moreover, the screening tool may be further improved by retraining and testing the algorithm on additional cohorts. To simplify the applicability of the screening tool, further studies may choose to measure the selected biomarkers using more widely available techniques such as ELISA. Possibly, IL12B can substitute IL12RB1 in the model, as IL12B was also differentially expressed in this study and assays may be more widely available. A limitation of the PEA technology is that the NPX values were not converted to an absolute concentration but could only be compared between samples in the same run. In this study, the healthy control samples were used to correct for batch effects between the testing and training cohorts, but this is not practical for use in the clinic. A solution would be to include standard curves for the biomarkers of interest, and/or to use a different technique such as ELISA in order to quantify these markers and identify cutoff values for the normal range.

After merging the two cohorts, differentially expressed proteins specific for CVIDid as a whole, and CVID with autoimmunity, GLILD and splenomegaly, were identified. In our study, IFNγ-responsive cytokines CXCL9, − 10 and − 11 were upregulated in CVIDid, which are instrumental in Th1 skewing [30]. This is in line with previous data showing accumulating support for Th1/T follicular helper (Tfh) 1 skewing in CVIDid patients [21, 31]. Our data also indicates Th17 activation in CVID patients with immune dysregulation, reflected by upregulation of IL17, IL12B (the subunit for both IL12 and IL23) and CCL20 in CVIDid, which are associated with Th17 skewing and consistent with reports in other inflammatory disorders [32]. Upregulation of IL6, which may be consistent with Th17 and Tfh activation, was observed in both CVIDio and CVIDid compared to HC in the present study. Other Th17-associated cytokines such as IL21 and IL22 were not measured here. However, these findings are in contrast to previous studies reporting a suppression of IL17/TH17 cells in CVID [18, 33, 34]. A possible alternative source for IL17 production in these patients are a population of innate lymphoid cells that produce IL17 and IFNγ that were described in the blood of CVID patients with immune dysregulation [35].

In parallel to the Th1/Th17-associated inflammatory cytokines, we observed an increase of immune regulatory proteins. The induction of IL10 often accompanies production of proinflammatory cytokines in both myeloid and T helper cells, suggestive of an (in this case insufficient) compensatory feedback loop that limits immune pathology [36]. While IL10 can be produced by regulatory T cells, there was no coupregulation of TGF-β in this cohort, suggesting an alternative source for the IL10, such as monocytes. Induction of IL10 due to persistent antigen exposure can result in functional T cell exhaustion [37]. Chronic antigen exposure in CVIDid despite IgRT may be related to bacterial translocation from the gastrointestinal tract [38].

LAG3, TNFRSF9 (also known as 4-1BB) and CD83 were also upregulated in immune dysregulation in our cohorts and may reflect a chronically activated immune state and immune exhaustion. LAG3 is a coinhibitory receptor that limits the proliferative capacity of T cells and can confer suppressive properties to other T cells [39, 40]. However, the soluble form of LAG3 has been shown to be immune potentiating and is being investigated as a vaccine adjuvant [41]. TNFRSF9 also has complex effects on different cell types [42, 43], and increased serum levels of soluble TNFRSF9 correlate with disease severity in rheumatoid arthritis and other autoimmune diseases, suggesting that the soluble form may act as a decoy receptor preventing TNFSF9-mediated suppression of T cells [44]. Similar dynamics have been described for antigen presenting cell maturation marker CD83, which is reported to be immunosuppressive in autoimmune diseases in its soluble form [25].

Taken together, the upregulation of IL10, LAG3, TNFRSF9, and CD83 in immune dysregulation in CVID may indicate a chronic and refractory immune activated state. Whether functional exhaustion of T cells also occurs in these patients will need to be assessed on a cellular level. One study that investigated this in a mixed cohort of CVIDid and CVIDio patients reported functional exhaustion of CD4 T cells, which correlated with serum endotoxemia [45]. Authors of this study did not find upregulation of membrane-bound LAG3, but do report increased surface expression of PD-1 [45]. In our study, serum levels of PD-L1 (the ligand for PD-1) were significantly increased in CVID with autoimmunity but did not pass the fold-change criterion (adj. *p* = 0.029, FC = 1.32).

To conclude, this study shows a promising first step in the development of a screening tool for immune dysregulation in CVID using serum proteins IL10, IL12RB1, and CD83 as biomarker. In addition, the immune dysregulation clinical phenotype was associated with increased levels of Th1- and Th17- related serum proteins, and displays a complex immune regulatory profile that includes IL10, LAG3, TNFRSF9, and CD83 signaling. Further research focusing on the dynamics of these biomarkers longitudinally is necessary to evaluate its use as an early detection screening tool for immune dysregulation in CVID. In addition, studying the behavior of these biomarkers under immunosuppressive therapy will indicate whether these markers can be used to monitor therapeutic response.

## Supplementary Information

 ESM 1: Supplementary figures and tables S2-S4 (DOCX 998 kb).

ESM 2: Supplementary table S1 (XLSX 250 kb).
