# Supplementary material for: Targeted Proteomics Reveals Inflammatory Pathways that Classify Immune Dysregulation in Common Variable Immunodeficiency
Source: J Clin Immunol. 2020 Nov 15;41(2):362–73. doi: 10.1007/s10875-020-00908-1 (PMC7858548; doi:10.1007/s10875-020-00908-1)
Supplement: Supplementary file 1 — (DOCX 998 kb). [file 10875_2020_908_MOESM1_ESM.docx]

**Supplementary data Serum Cytokines in CVID**


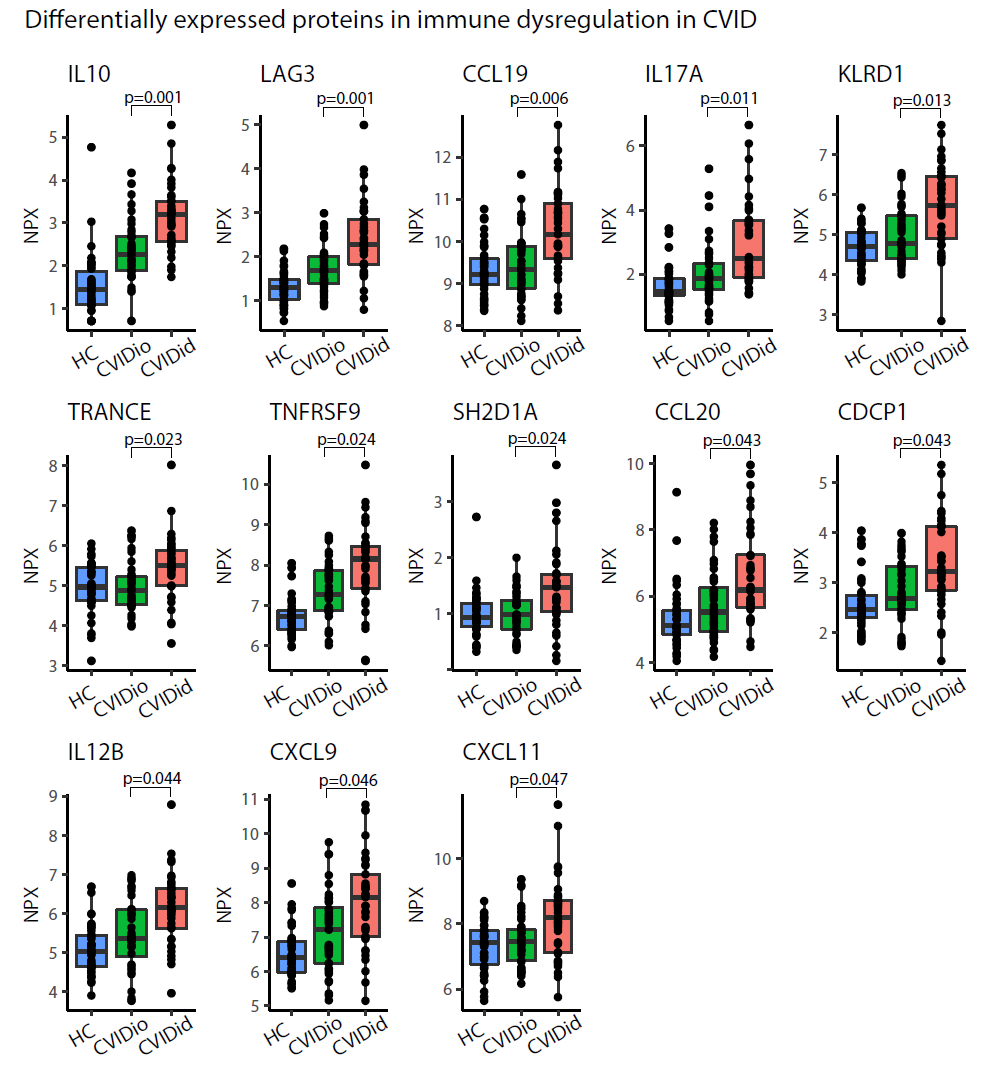


**Supplementary figure S1A**: Boxplots of differentially expressed proteins (log2 fold change >0.58 and FDR-adjusted p <0.05) in CVID with immune dysregulation (CVIDid, n=37) as compared to CVID with infection only CVIDio, n=40). The horizontal line inside the box represents the median. The whiskers represent the lowest and highest values within 1.5×interquartile range. P-values: Mann-Whitney U test after FDR correction.


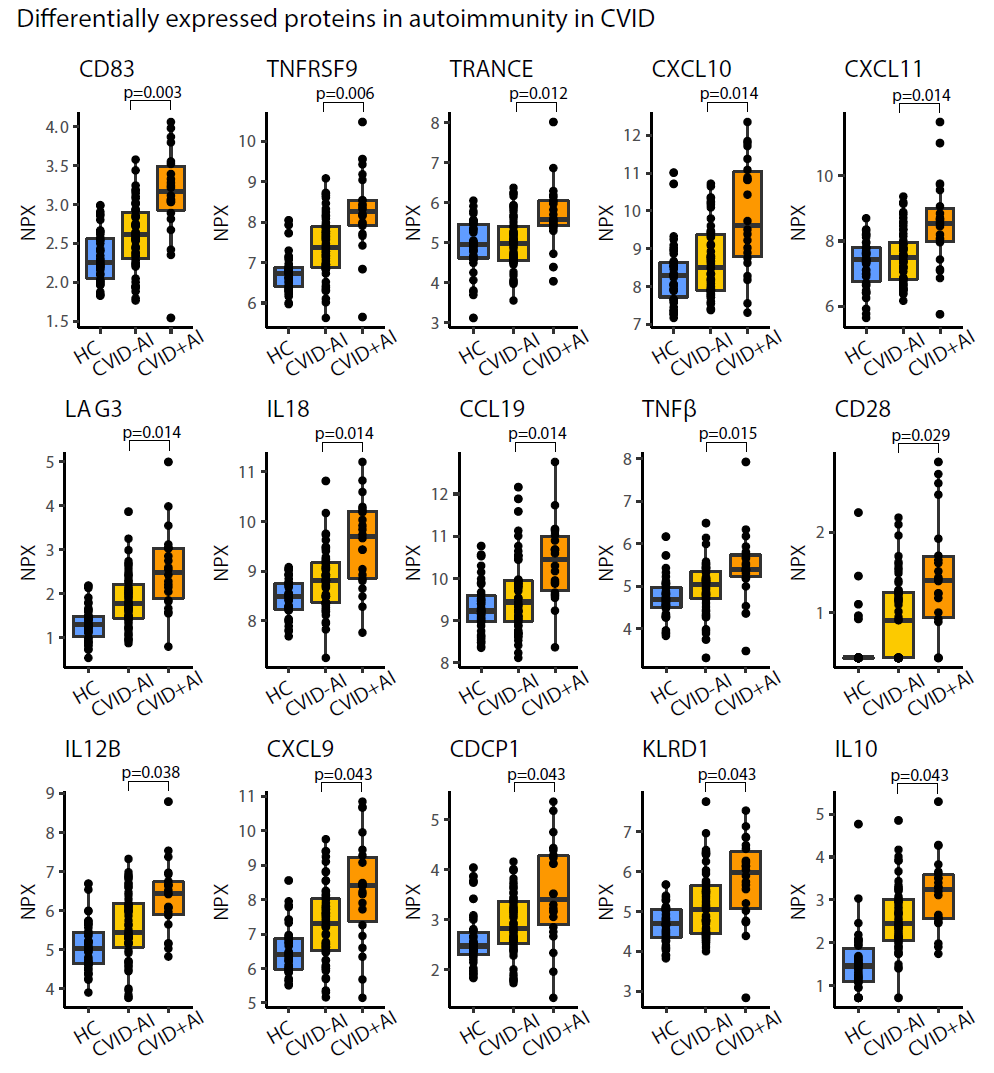


**Supplementary figure S1B**: Boxplots of differentially expressed proteins (log2 fold change >0.58 and FDR-adjusted p <0.05) upregulated in CVID with autoimmunity (n=22) as compared to CVID without autoimmunity (n=55). The horizontal line inside the box represents the median. The whiskers represent the lowest and highest values within 1.5×interquartile range. P-values: Mann-Whitney U test after FDR correction.


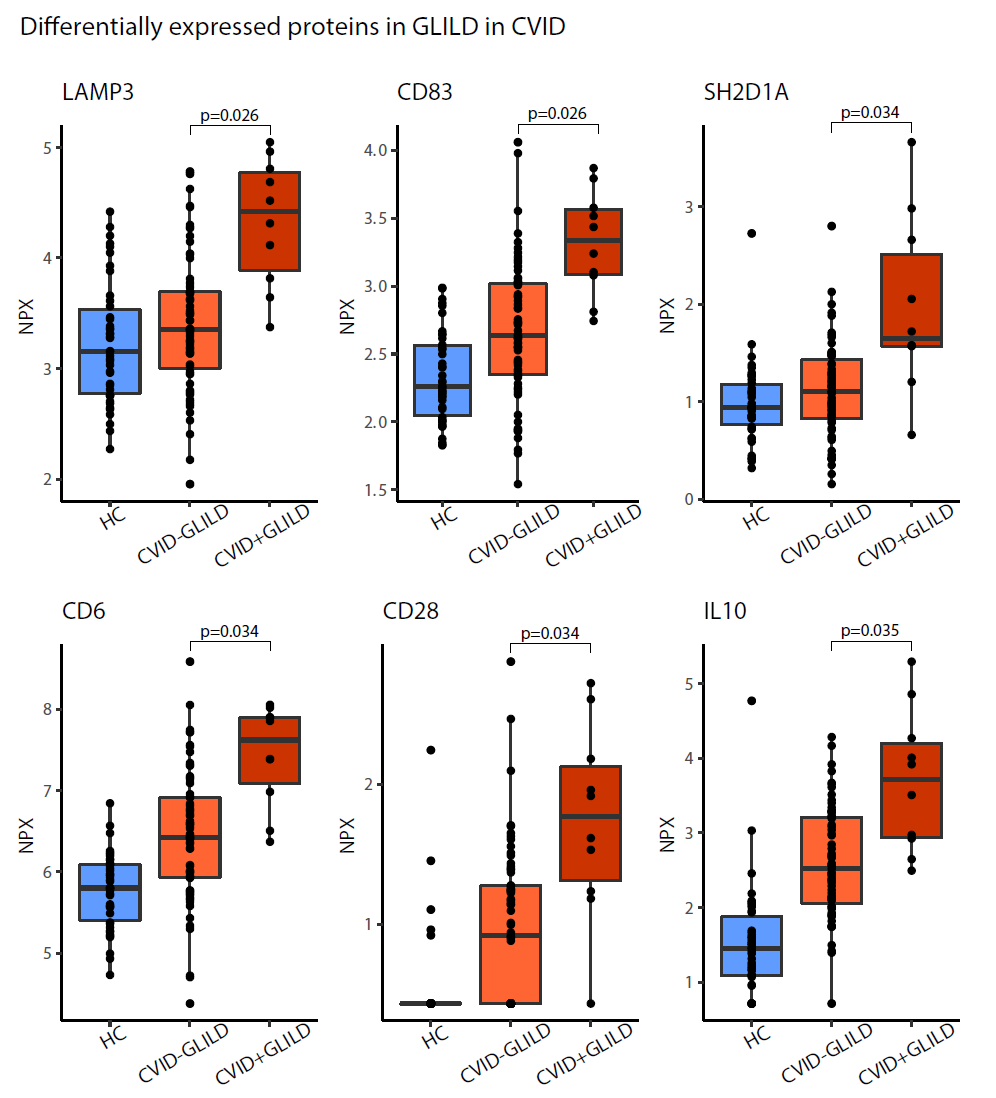


**Supplementary figure S1C**: Boxplots of differentially expressed proteins (log2 fold change >0.58 and FDR-adjusted p <0.05) upregulated in CVID with GLILD (n=10) as compared to CVID without GLILD (n=67). The horizontal line inside the box represents the median. The whiskers represent the lowest and highest values within 1.5×interquartile range. P-values: Mann-Whitney U test after FDR correction.


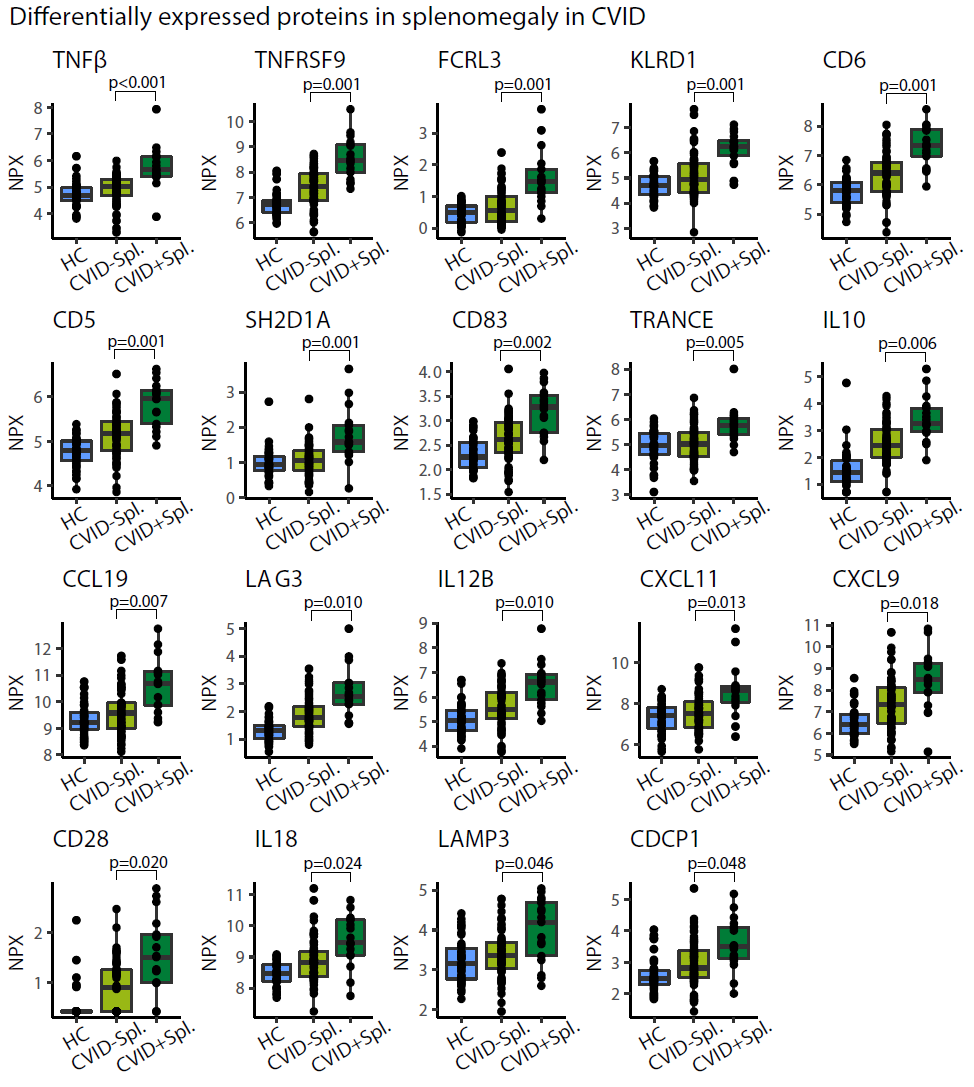


**Supplementary figure S1D**: Boxplots of differentially expressed proteins (log2 fold change >0.58 and FDR-adjusted p <0.05) upregulated in CVID with splenomegaly (CVID+Spl., n=17) as compared to CVID without splenomegaly (CVID-Spl., n=60). The horizontal line inside the box represents the median. The whiskers represent the lowest and highest values within 1.5×interquartile range. P-values: Mann-Whitney U test after FDR correction.


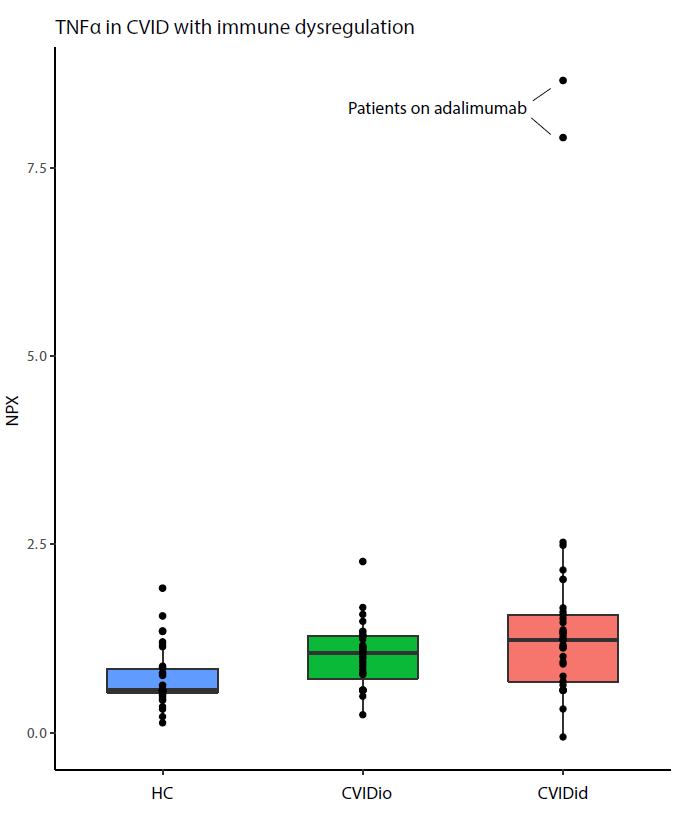


**Supplementary figure S2**: Boxplots of TNF-α in CVID with immune dysregulation (CVIDid, n=37) as compared to CVID with infection only CVIDio, n=40). The horizontal line inside the box represents the median. The whiskers represent the lowest and highest values within 1.5×interquartile range. P-values: Mann-Whitney U test after FDR correction.

**Supplementary table S1** has been added as a separate file.

Simplified biomarker list:

| 4E-BP1 | CLEC4C | FAM3B |  | IL-17C | LIF-R | PTH1R |
| --- | --- | --- | --- | --- | --- | --- |
| ADA | CLEC4D | FCRL3 |  | IL18 | LILRB4 | SCF |
| AREG | CLEC4G | FCRL6 |  | IL-18R1 | LY75 | SH2B3 |
| ARNT | CLEC6A | FGF-19 |  | IL2 | MASP1 | SH2D1A |
| ARTN | CLEC7A | FGF2 |  | IL-20 | MCP-1 | SIRT2 |
| AXIN1 | CNTNAP2 | FGF-21 |  | IL-20RA | MCP-2 | SIT1 |
| BACH1 | CSF-1 | FGF-23 |  | IL-22 RA1 | MCP-3 | SLAMF1 |
| Beta-NGF | CST5 | FGF-5 |  | IL-24 | MCP-4 | SPRY2 |
| BIRC2 | CX3CL1 | Flt3L |  | IL-2RB | MGMT | SRPK2 |
| BTN3A2 | CXADR | FXYD5 |  | IL33 | MILR1 | ST1A1 |
| CASP-8 | CXCL1 | GALNT3 |  | IL4 | MMP-1 | STAMPB |
| CCL11 | CXCL10 | GDNF |  | IL5 | MMP-10 | STC1 |
| CCL19 | CXCL11 | GLB1 |  | IL6 | NCR1 | TANK |
| CCL20 | CXCL12 | HCLS1 |  | IL7 | NF2 | TGF-alpha |
| CCL23 | CXCL5 | HEXIM1 |  | IL8 | NFATC3 | TNF |
| CCL25 | CXCL6 | HGF |  | IRAK1 | NRTN | TNFB |
| CCL28 | CXCL9 | HNMT |  | IRAK4 | NT-3 | TNFRSF9 |
| CCL3 | DAPP1 | HSD11B1 |  | IRF9 | NTF4 | TNFSF14 |
| CCL4 | DCBLD2 | ICA1 |  | ITGA11 | OPG | TPSAB1 |
| CD244 | DCTN1 | IFN-gamma |  | ITGA6 | OSM | TRAF2 |
| CD28 | DDX58 | IFNLR1 |  | ITGB6 | PADI2 | TRAIL |
| CD40 | DFFA | IL-1 alpha |  | ITM2A | PD-L1 | TRANCE |
| CD5 | DGKZ | IL10 |  | JUN | PIK3AP1 | TREM1 |
| CD6 | DNER | IL-10RA |  | KLRD1 | PLXNA4 | TRIM21 |
| CD83 | DPP10 | IL-10RB |  | KPNA1 | PPP1R9B | TRIM5 |
| CD8A | EDAR | IL-12B |  | KRT19 | PRDX1 | TSLP |
| CDCP1 | EGLN1 | IL12RB1 |  | LAG3 | PRDX3 | TWEAK |
| CDSN | EIF4G1 | IL13 |  | LAMP3 | PRDX5 | uPA |
| CKAP4 | EIF5A | IL-15RA |  | LAP TGF-beta-1 | PRKCQ | VEGFA |
| CLEC4A | EN-RAGE | IL-17A |  | LIF | PSIP1 | ZBTB16 |

**Supplementary table S2**: results of genetic screening in training and testing cohort. VUS=variants of unknown significance

| **VUS found in training cohort** | **Pathogenic mutations found in training cohort** |
| --- | --- |
| CVIDid: Heterozygote JAK3, VUS in PLCG2 | CVIDio: TNFRSF13B (TACI) |
| CVIDid: Heterozygote UNC13D | CVIDid: CTLA4 |
|  | CVIDid: STAT1 GOF |
| **VUS found in testing cohort** | **Pathogenic mutations found in testing cohort** |
| CVIDid: PRKDC | CVIDid: TNFRSF13B (3x) |
|  | CVIDid: PIK3R1 |

**Supplementary table S3A**: Performance of random forest, elastic net and extreme gradient boosting algorithms trained on training data. Area under the curve (AUC) based on Receiver-Operator Curves of classification performance on testing cohort. Sensitivity and specificity based on threshold selected for maximum Youden's Index.

|  | Random Forest | ElasticNet | ExtremeGradientBoosting |
| --- | --- | --- | --- |
| AUC | 0.71 | 0.72 | 0.71 |
| Sensitivity | 0.70 | 0.74 | 0.78 |
| Specificity | 0.71 | 0.71 | 0.67 |

**Supplementary table S3B**: standardized regression coefficients of the elastic net algorithm

| **ElasticNet** | **standardized regression coefficients** |
| --- | --- |
| MILR1 | 49.5275225 |
| LILRB4 | 45.9481322 |
| IL10 | 39.4471273 |
| IL12RB1 | 32.5195959 |
| CXCL10 | 26.8637128 |
| BTN3A2 | 23.6616556 |
| CD83 | 22.3648257 |
| IL.17A | 22.1304286 |
| CCL19 | 20.07503 |
| CXCL5 | 19.4975474 |
| CLEC6A | 18.9570038 |
| LAG3 | 18.6745838 |
| IL5 | 18.0662128 |
| FCRL6 | 17.8391726 |
| CXCL11 | 16.1581789 |
| CCL20 | 14.5387933 |
| IL18 | 13.3605353 |
| SH2D1A | 9.0432834 |
| CD28 | 8.8901486 |
| CKAP4 | 2.8303555 |
| CXCL9 | 1.4464496 |
| ITM2A | 0.9145627 |
| KLRD1 | 0.1589813 |

**Supplementary table S3C**: standardized regression coefficients of the extreme gradient boosting algorithm

| **ExtremeGradientBoosting** | **standardized regression coefficients** |
| --- | --- |
| MILR1 | 46.19873132 |
| CD83 | 24.38600922 |
| LILRB4 | 22.38396687 |
| IL10 | 16.60761097 |
| CLEC6A | 10.40267680 |
| IL12RB1 | 9.87841521 |
| CCL25 | 8.86002056 |
| DCBLD2 | 8.42205461 |
| TRANCE | 6.53458466 |
| CCL20 | 6.44588911 |
| ITM2A | 5.74735616 |
| SH2D1A | 4.86901403 |
| IL18 | 4.57302688 |
| PSIP1 | 4.51630353 |
| BTN3A2 | 4.39895158 |
| CD244 | 3.16099943 |
| KLRD1 | 2.44351124 |
| CXCL10 | 2.08361928 |
| CD28 | 2.02518904 |
| FGF.21 | 1.95971723 |
| NCR1 | 1.81192499 |
| NFATC3 | 1.75484145 |
| CXCL11 | 1.67102412 |
| FCRL6 | 1.49078755 |
| X4E.BP1 | 1.27968655 |
| CKAP4 | 1.27881171 |
| KRT19 | 1.03650139 |
| MMP.1 | 1.01803351 |
| HNMT | 0.89113541 |
| CCL19 | 0.84695373 |
| CXADR | 0.84156278 |
| IL5 | 0.82510504 |
| CCL11 | 0.82376997 |
| Beta.NGF | 0.82260374 |
| CD5 | 0.79428321 |
| BACH1 | 0.78728905 |
| FGF.19 | 0.68545008 |
| CLEC4G | 0.67419116 |
| TNFB | 0.64908048 |
| TRIM5 | 0.63198289 |
| IL.17C | 0.63148997 |
| IL7 | 0.61091238 |
| TANK | 0.60665911 |
| CXCL5 | 0.59876505 |
| TGF.alpha | 0.53295115 |
| GALNT3 | 0.53198514 |
| ZBTB16 | 0.51442105 |
| CCL28 | 0.4742683 |
| DNER | 0.21160083 |
| IFNLR1 | 0.20355936 |
| MCP.3 | 0.20194989 |
| GDNF | 0.19465451 |
| PIK3AP1 | 0.19146449 |
| ITGA6 | 0.19084172 |
| CDSN | 0.18973834 |
| TNF | 0.18456823 |
| MASP1 | 0.18163372 |
| DPP10 | 0.17539809 |
| MCP.2 | 0.17471131 |
| HEXIM1 | 0.17235877 |
| IL.10RA | 0.16277171 |
| PTH1R | 0.16087889 |
| HSD11B1 | 0.1604582 |
| EIF4G1 | 0.16014179 |
| EDAR | 0.15683554 |
| CST5 | 0.15533562 |
| PLXNA4 | 0.14730777 |
| CLEC7A | 0.14674432 |
| LAG3 | 0.13184895 |
| CXCL9 | 0.12793841 |
| IFN.gamma | 0.1270753 |
| CCL4 | 0.1263282 |
| MGMT | 0.1117052 |
| ITGA11 | 0.09234367 |
| NT.3 | 0.0868073 |
| CCL3 | 0.08641593 |
| LAP.TGF.beta.1 | 0.08193079 |
| CLEC4A | 0.07763499 |
| EN.RAGE | 0.07434307 |
| TRAIL | 0.07028783 |
| LIF | 0.06844958 |
| IL8 | 0.06356907 |

**Supplementary table S4**: post-hoc analysis assessing the prediction accuracy of reduced combinations of the classifying model on the testing cohort. AUC = area under the curve

|  | **AUC** | **sensitivity** | **specificity** |
| --- | --- | --- | --- |
| **IL10+IL12RB1+CD83** | 0.765 | 0.786 | 0.708 |
| **IL10+IL12RB1** | 0.772 | 0.739 | 0.750 |
| **IL10+CD83** | 0.741 | 0.780 | 0.708 |
| **IL12RB1+CD83** | 0.748 | 0.739 | 0.625 |
| **IL10** | 0.743 | 0.826 | 0.625 |
| **IL12RB1** | 0.730 | 0.870 | 0.500 |
| **CD83** | 0.728 | 0.826 | 0.583 |
